# Case Report: Endoscopic submucosal dissection and endoscopic transcecal appendectomy for synchronous sigmoid and appendiceal adenomas with calcified Schistosoma eggs

**DOI:** 10.3389/fonc.2025.1684500

**Published:** 2025-10-01

**Authors:** Yu Zhou, Liying Zhu, Duoshan Niu, Li Xu, Xiaowu Xu, Xingxing Kang, Yemei Du, Daoxing He

**Affiliations:** ^1^ Department of Gastroenterology, Xuancheng People’s Hospital, Affiliated Xuancheng Hospital Wannan Medical College, Xuancheng, Anhui, China; ^2^ Department of Hematology, The First Affiliated Hospital of Nanjing Medical University, Jiangsu Province Hospital, Nanjing, China; ^3^ Department of Pathology, Xuancheng People’s Hospital, Affiliated Xuancheng Hospital Wannan Medical College, Xuancheng, Anhui, China; ^4^ Department of Imaging, Xuancheng People’s Hospital, Affiliated Xuancheng Hospital Wannan Medical College, Xuancheng, Anhui, China

**Keywords:** Schistosoma haematobium egg, natural orifice transluminal endoscopic surgery, appendiceal tumor, endoscopic submucosal dissection, endoscopic transcecal appendectomy

## Abstract

**Background:**

Schistosomiasis is an endemic parasitic disease still prevalent in some regions of China. Chronic infection may cause recurrent inflammation and intestinal mucosal remodeling, increasing the risk of lesions such as adenomas. Although intestinal lesions associated with schistosomiasis have been reported, cases involving both the colon and appendix with egg deposition are rare. With the maturation of natural orifice transluminal endoscopic surgery (NOTES), endoscopic transcecal appendectomy (ETA), a specific NOTES technique, has been increasingly applied in clinical practice. In this case, two lesions in the colon and appendix were completely resected using endoscopic submucosal dissection (ESD) followed by ETA, offering a novel approach for the minimally invasive treatment of complex lesions.

**Case presentation:**

A 77-year-old woman presented with hematochezia. Colonoscopy revealed a bulging lesion in the sigmoid colon obstructing the passage of the scope. After complete resection of the lesion by ESD, the scope was advanced to the ileocecum, where a tumor-like lesion protruding from the appendiceal orifice was identified. ETA was subsequently performed with pathologic and imaging confirmation.

**Conclusion:**

This case suggests that Schistosoma haematobium infection may contribute to intestinal epithelial neoplasia. The combination of ESD and ETA offers a safe and feasible minimally invasive strategy for the treatment of concurrent colonic and appendiceal lesions.

## Introduction

Schistosomiasis is widely distributed in the Middle East, Southeast Asia, and sub-Saharan Africa, and was once highly endemic in China’s Yangtze River basin (1), Despite the remarkable success of control efforts and the significant decline in clinical infections, chronic intestinal lesions caused by worm egg deposition can still be observed in elderly patients (2). Schistosoma haematobium has been associated with colorectal neoplasia (3, 4), and egg deposition may induce or promote intestinal epithelial neoplasia through mechanisms such as chronic mucosal inflammation, tissue fibrosis, and alterations in the local microenvironment (5).

Traditionally, due to its unique anatomical structure, lesions of the appendix have been managed through surgical resection. In recent years, with the rapid advancement of endoscopic technology, natural orifice transluminal endoscopic surgery has been gradually introduced into the clinical management of gastrointestinal diseases. However, controversies remain, particularly regarding the technical difficulty of treating appendiceal lesions (6), Endoscopic transcecal appendectomy, as a specific NOTES technique, has demonstrated feasibility and safety in several studies (7–9). In this paper, we report the case of an elderly female patient who presented with hematochezia. Colonoscopy revealed adenomatous lesions in both the sigmoid colon and the appendix, which were completely resected using ESD and ETA, respectively. Postoperative pathology confirmed the presence of calcified Schistosoma eggs in both lesions. This case not only suggests that chronic Schistosoma haematobium infection may contribute to the development of intestinal neoplasia, but also highlights the potential of ESD combined with ETA for treating lesions in anatomically complex regions.

## Case description

A 77-year-old female patient was admitted to our hospital with a 10-day history of hematochezia. The blood was dark red, not mixed with feces, and there was no associated anal pain, abdominal pain, or significant weight loss. She had a history of hypertension, controlled with oral amlodipine besylate, and denied a history of other chronic conditions such as diabetes mellitus.

**Figure 1:**
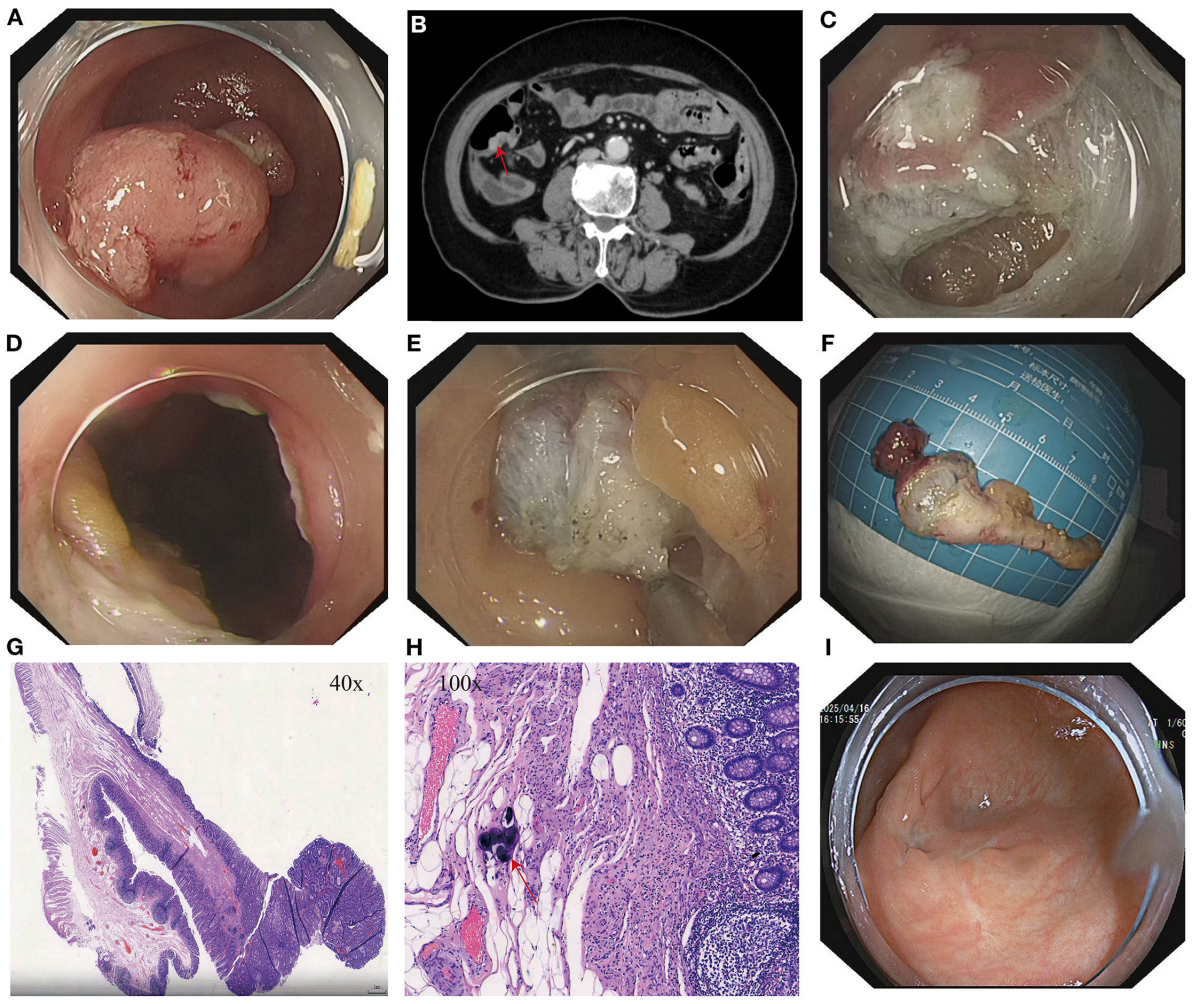
**(A)** Lesion at the appendiceal orifice. **(B)** Contrast-enhanced abdominal CT showing a nodular lesion in the cecum with a tegument-like structure extending into the appendix (red arrow). **(C)** Intraoperative incision during ETA procedure. **(D)** Entry into the abdominal cavity during ETA. **(E)** Dissection of the appendix and surrounding tissues. **(F)** Resected appendiceal specimen following ETA. **(G)** Histopathological findings of the appendiceal lesion. **(H)** Calcified *Schistosoma haematobium* eggs (red arrows) observed under high-power magnification in the appendix following ETA. **(I)** Follow-up colonoscopy at 15 months showing a well-healed surgical scar at the cecum with no evidence of recurrence or stenosis.

**Figure 2:**
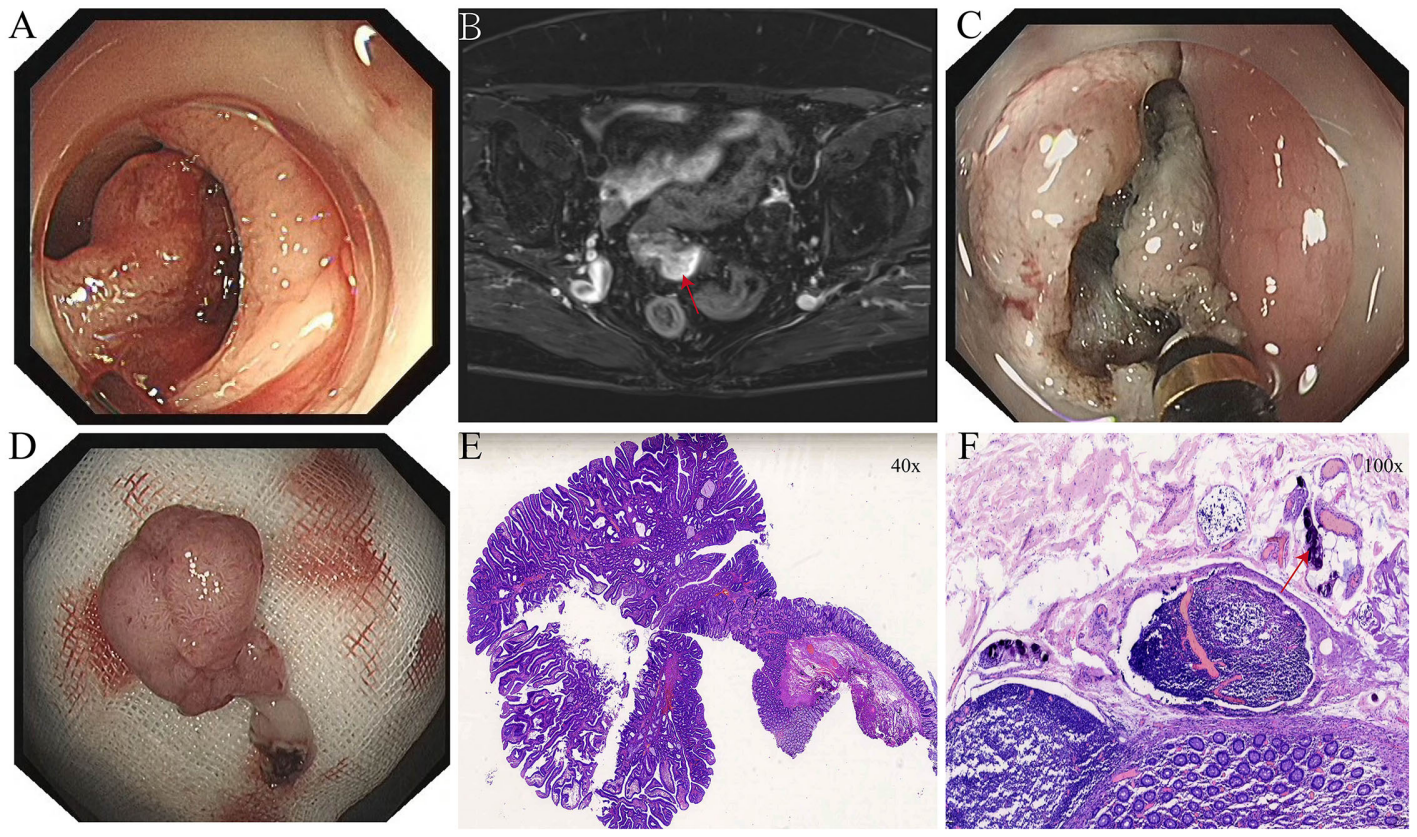
**(A)** Elevated lesion in the sigmoid colon obstructing the intestinal lumen. **(B)** Pedunculated neoplasm in the sigmoid colon as seen on pelvic MRI with contrast enhancement (red arrow). **(C)** Intraoperative incision of the elevated sigmoid colon lesion. **(D)** Resected sigmoid colon specimen following ESD. **(E)** Histopathological findings of the sigmoid colon lesion after ESD. **(F)** Calcified *Schistosoma haematobium* eggs (red arrows) observed in the sigmoid colon under high-power magnification.

After admission, relevant examinations were completed. Routine blood tests, coagulation profile, tumor markers (CEA, AFP, CA125, CA15-3, CA19-9), and thyroid function were all within normal limits. Tumor markers were measured as part of the initial workup for unexplained hematochezia, but they served only as auxiliary tests, with the final diagnosis relying on endoscopy and histopathology. Biochemical tests revealed elevated gamma-glutamyl transferase (GGT) at 74.6 U/L and blood glucose (GLU) at 6.23 mmol/L; all other parameters were within normal limits. Electrocardiogram (ECG) showed sinus rhythm with no abnormalities. Abdominal and pelvic CT revealed hypodense foci in the right hepatic lobe and both kidneys, a mass in the left adnexal region, and mild thickening of the anal canal wall. Contrast-enhanced abdominopelvic CT revealed a mass in the left adnexal region, possibly an ovarian fibroma or theca cell tumor. A nodular lesion was observed in the cecum, with a visible tubule-like structure extending into the appendix, suggestive of an adenoma (**Figure 1B**). Gastroscopy revealed chronic atrophic gastritis with erosions (C-2), consistent with current Helicobacter pylori infection. Colonoscopy revealed a bulging lesion in the sigmoid colon obstructing the lumen (**Figure 2A**). The scope could not be advanced further. A biopsy was performed, and pathology suggested an adenomatous polyp. Pelvic MRI with contrast revealed a luminal mass in the sigmoid colon, possibly an adenoma (**Figure 2B**), and showed multiple uterine fibroids.

After thorough evaluation confirmed no contraindications to endoscopic surgery, the patient underwent ESD of the sigmoid colon tumor (**Figure 2**). The lesion was completely resected, and the endoscope was able to smoothly advance through the previous site to reach the ileocecal region. At the appendiceal orifice, a tumor-like lesion protruding from the lumen was identified, biopsied, and sent for pathological examination (**Figure 1A**). Postoperative pathology of the sigmoid colon lesion confirmed a tubular adenoma, with calcified Schistosoma egg deposits in the submucosal layer at the tip (**Figure 2F**). The biopsy of the appendiceal lesion indicated adenomatous changes.

After the procedure, hematochezia resolved, and the patient’s clinical symptoms improved. However, the lesion at the appendiceal orifice showed adenomatous changes and required further intervention. The conventional treatment approach—surgical resection of the appendix with ileocecal and partial small bowel resection—is highly invasive, associated with delayed postoperative recovery, and carries risks such as abdominal wall infection, postoperative pain, incisional hernia, and prolonged hospitalization, especially in elderly patients (10–12). After thorough communication with the patient and her family, and obtaining informed consent, our team performed ETA, achieving complete resection of the appendix and associated lesion (**Figure 1**). Postoperatively, the patient was managed with two days of fasting, bed rest, anti-infective therapy, and symptomatic supportive care. Recovery was uneventful.

Postoperative pathology confirmed that the appendiceal orifice lesion was an adenomatous polyp with high-grade intraepithelial neoplasia. Calcified Schistosoma eggs and chronic inflammatory changes were observed in the mucosa at the tip (**Figure 1H**). The patient experienced no postoperative abdominal pain, hematochezia, or fever, and was discharged one week after surgery in good general condition. Physical examination revealed no abdominal tenderness or rebound pain.

A colonoscopy performed 15 months after surgery revealed a well-healed surgical scar in the ileocecal region, with no evidence of recurrence or intestinal stenosis (**Figure 1I**).

## Discussion

In this case, the patient presented with hematochezia, and colonoscopy revealed a neoplastic lesion in the sigmoid colon that obstructed the intestinal lumen. Following complete resection of the lesion via ESD, the endoscope was able to advance to the cecum, where an unexpected neoplastic lesion was discovered at the appendiceal orifice. ETA was subsequently performed to resect the appendix and the lesion entirely. Pathological examination confirmed adenomatous polyps in both the sigmoid colon and appendix, with high-grade intraepithelial neoplasia noted in the appendiceal lesion. Notably, both lesions contained calcified *Schistosoma haematobium* eggs.

This case is characterized by the following distinctive features: 1. Bilateral distribution of neoplastic lesions, located separately in the sigmoid colon and appendix; 2. Presence of calcified schistosome eggs in both lesions, raising the possibility that chronic *Schistosoma* infection may contribute to the development of colorectal neoplasia (4);3. Successful application of endoscopic transcecal appendectomy, which allowed complete resection of the appendiceal lesion while avoiding the surgical trauma associated with conventional laparoscopic surgery, particularly relevant for elderly patients (9, 13). In summary, this case offers unique insights in terms of potential etiological associations, rare lesion distribution, and minimally invasive treatment strategies, providing valuable clinical reference.

Schistosomiasis is a chronic parasitic disease characterized by egg deposition in host tissues, leading to chronic granulomatous inflammation, fibrosis, and structural disruption of the intestinal mucosa (4, 14). Accumulating evidence suggests that schistosome egg antigens may trigger localized immune responses, oxidative stress, DNA damage, and epigenetic modifications, ultimately activating oncogenic signaling pathways (5). Epidemiological studies have demonstrated a significantly higher incidence of adenomatous lesions and colorectal carcinoma in schistosome-infected populations compared to non-infected individuals (15, 16). The appendix, given its narrow lumen and anatomical structure, is particularly prone to egg deposition and chronic inflammation (16–18). In this patient, calcified schistosome eggs were observed in both the sigmoid colon and appendix. This supports the hypothesis that chronic schistosome infection may promote epithelial hyperplasia and neoplastic transformation at multiple intestinal sites, potentially contributing to colorectal tumorigenesis.

Historically, Schistosoma-associated colorectal tumors have predominantly involved the rectum and sigmoid colon, with adenocarcinoma and mucinous adenocarcinoma being the most common pathological subtypes (2, 4). While occasional case reports have documented schistosome eggs in appendiceal adenomas, such occurrences are exceedingly rare (17). To our knowledge, there are no previously published cases describing synchronous adenomatous lesions in both the sigmoid colon and appendix with concurrent schistosomal egg deposition in both sites, highlighting the rarity and significance of this case.

NOTES is a novel, minimally invasive surgical approach that utilizes natural orifices to access the peritoneal cavity, thereby avoiding external incisions. This technique offers potential advantages including reduced postoperative pain, lower risk of incisional complications, and faster recovery (8, 19). With advancements in endoscopic instruments and operative techniques, NOTES has been gradually adopted in selected procedures, such as appendectomy and certain hepatobiliary surgeries—particularly in elderly patients, those with significant comorbidities, or patients who are poor candidates for conventional surgery (19–23). In the present case, the patient’s advanced age and anatomical involvement rendered traditional laparoscopic appendectomy challenging, as it would have required resection of the ileocecal segment and part of the small intestine (10, 12). Instead, ETA enabled complete resection of the lesion with minimal trauma, shortened hospitalization, and excellent postoperative recovery, thus demonstrating the feasibility and advantages of NOTES in managing appendiceal lesions in complex scenarios.

Although the detection of calcified *Schistosoma* eggs in both lesions suggests a potential role in tumorigenesis, definitive conclusions regarding causality remain premature. Critical questions—such as the viability of the eggs, local immune microenvironment dynamics, and molecular pathways involved—require further investigation through mechanistic studies. Additionally, while ETA represents a promising application of NOTES, it remains technically demanding and is still in the early stages of clinical adoption. Large-scale, multicenter studies are warranted to validate its safety, efficacy, and long-term outcomes in the management of appendiceal lesions.

## Conclusion

This case of synchronous adenomatous lesions in the sigmoid colon and appendix, both accompanied by calcified *Schistosoma* egg deposition, suggests a potential association between chronic schistosomal infection and intestinal neoplasia. The complete removal of both lesions using a combination of ESD and ETA highlights the safety, efficacy, and minimally invasive advantages of this approach, particularly in elderly patients. This case not only enhances clinical awareness of the long-term complications of schistosomiasis but also underscores the practical value and future potential of NOTES in the management of complex gastrointestinal lesions.

## Data Availability

The original contributions presented in the study are included in the article/supplementary material. Further inquiries can be directed to the corresponding author.

## References

[1] Colley DG Bustinduy AL Secor WE King CH . Human schistosomiasis. Lancet. (2014) 383:2253–64. doi: 10.1016/S0140-6736(13)61949-2, PMID: 24698483 PMC4672382

[2] Yang Y Wang X-Y Duan C Wang Z-J Sheng H-Y Xu X-L . Clinicopathological characteristics and its association with digestive system tumors of 1111 patients with Schistosomiasis japonica. Sci Rep. (2023) 13:15115. doi: 10.1038/s41598-023-42456-9, PMID: 37704736 PMC10500003

[3] Almeida GFG Freitas MALD Lira MMM Domingues ALC Neares L Silva LVMD . ASQO - Advanced Support for Quality-of-life in Oncology. Clinicopathological description of a subset of schistosoma mansoni associated colorectal cancer patients in an endemic area in Brazil. JCO. (2019) 37:e15154–4. doi: 10.1200/jco.2019.37.15_suppl.e15154

[4] Hamid HKS . Schistosoma japonicum–associated colorectal cancer: A review. Am J Trop Med Hyg. (2019) 100:501–5. doi: 10.4269/ajtmh.18-0807, PMID: 30560774 PMC6402928

[5] Weglage J Wolters F Hehr L Lichtenberger J Wulz C Hempel F . Schistosoma mansoni eggs induce Wnt/β-catenin signaling and activate the protooncogene c-Jun in human and hamster colon. Sci Rep. (2020) 10:22373. doi: 10.1038/s41598-020-79450-4, PMID: 33361772 PMC7758332

[6] Chen T Xu A Lian J Chu Y Zhang H Xu M . Transcolonic endoscopic appendectomy: a novel natural orifice transluminal endoscopic surgery (NOTES) technique for the sessile serrated lesions involving the appendiceal orifice. Gut. (2021) 70:1812–4. doi: 10.1136/gutjnl-2020-323018, PMID: 33483328 PMC8458066

[7] Ichkhanian Y Barawi M Seoud T Thakkar S Kothari TH Halabi ME . Endoscopic full-thickness resection of polyps involving the appendiceal orifice: a multicenter international experience. Endoscopy. (2022) 54:16–24. doi: 10.1055/a-1345-0044, PMID: 33395714

[8] Mueller J Kuellmer A Schiemer M Thimme R Schmidt A . Current status of endoscopic full-thickness resection with the full-thickness resection device. Dig Endosc. (2023) 35:232–42. doi: 10.1111/den.14425, PMID: 35997598

[9] Guo L Ye L Feng Y Bethge J Yang J Schreiber S . Endoscopic transcecal appendectomy: a new endotherapy for appendiceal orifice lesions. Endoscopy. (2022) 54:585–90. doi: 10.1055/a-1675-2625, PMID: 34905794 PMC9132730

[10] Turgut HT Subasi O . Comparison of ileocecal resection and right hemicolectomy in the surgical treatment of complicated appendicitis. Ulus Travma Acil Cerrahi Derg. (2023) 29:705–9. doi: 10.14744/tjtes.2023.83357, PMID: 37278071 PMC10315939

[11] Kobayashi T Hidaka E Ando A Koganezawa I Nakagawa M Yokozuka K . Preoperative scoring system for prediction of extended resection during emergency surgery for acute appendicitis. Langenbecks Arch Surg. (2023) 408:443. doi: 10.1007/s00423-023-03183-x, PMID: 37987920

[12] Monrabal Lezama M Álvarez Jurado MG Bras Harriott C Casas MA Schlottmann F . Beyond appendectomy: predictive factors for major resections in adult patients with acute appendicitis. World J Surg. (2025) 49:1466–70. doi: 10.1002/wjs.12588, PMID: 40387179

[13] Li X-Y Zhang D-F He M-J Li Q-L Zhou P-H . Endoscopic transcecal appendectomy for a laterally spreading tumor of the appendiceal stump. Endoscopy. (2025) 57:E84–5. doi: 10.1055/a-2512-3926, PMID: 39889774 PMC11785432

[14] Mohamed AR al Karawi M Yasawy MI . Schistosomal colonic disease. Gut. (1990) 31:439–42. doi: 10.1136/gut.31.4.439, PMID: 2110925 PMC1378420

[15] Wang Z Du Z Liu Y Wang W Liang M Zhang A . Comparison of the clinicopathological features and prognoses of patients with schistosomal and nonschistosomal colorectal cancer. Oncol Lett. (2020) 19:2375–83. doi: 10.3892/ol.2020.11331, PMID: 32194737 PMC7039146

[16] Abe T Yunokizaki H Iijima H Tamura K Lee ZL Higashi D . Schistosoma japonicum showing flat colon polyp appearance. Gastrointest Endosc. (2011) 73:820–2. doi: 10.1016/j.gie.2010.10.038, PMID: 21195405

[17] Nebel OT El Masry NA Castell DO Farid Z Fornes MF Sparks HA . Schistosomal disease of the colon: A reversible form of polyposis. Gastroenterology. (1974) 67:939–43. doi: 10.1016/S0016-5085(19)32747-7, PMID: 4426495

[18] Chen MC Chang PY Chuang CY Chen YJ Wang FP Tang YC . Colorectal cancer and schistosomiasis. Lancet (London England). (1981) 1:971–973. 6112388

[19] Bronzwaer MES Bastiaansen BAJ Koens L Dekker E Fockens P . Endoscopic full-thickness resection of polyps involving the appendiceal orifice: a prospective observational case study. Endosc Int Open. (2018) 6:E1112–9. doi: 10.1055/a-0635-0911, PMID: 30211300 PMC6133683

[20] Wu Z-W Ding C-H Song Y-D Cui Z-C Bi X-Q Cheng B . Colon sparing endoscopic full-thickness resection for advanced colorectal lesions: is it time for global adoption? Front Oncol. (2022) 12:967100. doi: 10.3389/fonc.2022.967100, PMID: 35912240 PMC9327091

[21] Hammoud GM Quick J Samiullah S Rao D Ibdah JA . Endoscopic full-thickness resection of a long intussuscepted appendix by use of a colonoscope. VideoGIE. (2019) 4:34–6. doi: 10.1016/j.vgie.2018.10.001, PMID: 30623159 PMC6317908

[22] Cai M-Y Martin Carreras-Presas F Zhou P-H . Endoscopic full-thickness resection for gastrointestinal submucosal tumors. Dig Endosc. (2018) 30 Suppl 1:17–24. doi: 10.1111/den.13003, PMID: 29658639

[23] Wang K Gao P Cai M Song B Zhou P . Endoscopic full-thickness resection, indication, methods and perspectives. Dig Endosc. (2023) 35:195–205. doi: 10.1111/den.14474, PMID: 36355358

